# Gamma Irradiation-Induced Changes in Microstructure of Cyclic Olefin Copolymer (COC) Revealed by NMR and SAXS Characterization

**DOI:** 10.3390/polym17131751

**Published:** 2025-06-24

**Authors:** Fan Zhang, Heng Lei, Feng Guo, Jiangtao Hu, Haiming Liu, Qing Wang, Weihua Liu, Zhe Xing, Guozhong Wu

**Affiliations:** 1 Shanghai Institute of Applied Physics, Chinese Academy of Sciences, No. 2019 Jialuo Road, Jiading District, Shanghai 201800, China; 2 University of Chinese Academy of Sciences, Beijing 100049, China; 3 School of Physical Science and Technology, ShanghaiTech University, Shanghai 201210, China

**Keywords:** cyclic olefin copolymer, microstructure, gamma radiation, NMR, SAXS

## Abstract

Cyclic Olefin Copolymer (COC) is an amorphous thermoplastic polymer synthesized through the catalytic copolymerization of α-olefin and cyclic olefin. When used in pre-filled syringes and pharmaceutical packaging, COCs require radiation sterilization. The radiation sterilization alters the microstructure of COC, which ultimately affects its performance and biosafety. In this study, to investigate the effects of γ-radiation on COC microstructures, ethylene-norbornene copolymers with various compositions, representative of COC, are studied by nuclear magnetic resonance (NMR) and small angle X-ray scattering (SAXS) techniques. During irradiation, the COC containing 35 mol% norbornene produced free radicals that triggered migration and reaction processes, leading to the formation of entanglements within flexible chain segments. This, in turn, affected nearby ring structures with high steric hindrance, resulting in a 9.2% decrease in internal particle size and an increase in particle spacing. Conversely, when the norbornene content in COC was increased to 57 mol%, the internal particle size increased by 17.9%, while the particle spacing decreased.

## 1. Introduction

Cyclic Olefin Copolymer (COC) is a class of polymers synthesized through the catalytic addition polymerization of norbornene or its derivatives as the primary monomer, combined with α-olefins as comonomers [1,2,3]. COCs are high-quality, transparent thermoplastic engineering plastics. Among these, ethylene-norbornene copolymers have gained significant attention due to their appealing properties and were commercialized under the trade name TOPAS by German Ticona in 2000. The properties of these materials can be finely turned by adjusting the molar ratio of norbornene during polymerization. These innovative commercial materials boast a unique combination of performance advantages, including variable glass transition temperatures ranging from 65 °C to 178 °C, heat resistance, chemical resistance to common solvents, low moisture absorption, high water barrier properties, excellent mechanical strength, ease of extrusion and thermoforming, compatibility with polyolefin, and exceptional biocompatibility and inertia. These numerous benefits have led to their widespread application in medical devices and packaging materials, for which COCs must undergo rigorous sterilization processes to ensure sterility and safety. γ-Ray sterilization is one of the most commonly used methods, as commercially successful technologies have been developed that enable the rapid sterilization of a wide range of disposable medical devices. However, the effects of irradiation sterilization on the properties of polymer materials are unavoidable. The high-energy electrons generated during the irradiation process are known to create reaction intermediates and free radicals within the polymer, which can follow multiple reaction pathways, leading to cross-linking or cleavage [4,5,6,7]. These changes can alter the mechanical and surface properties of the polymer. In previous research, we investigated the types of free radicals produced in COC upon exposure to gamma radiation and examined the mechanisms of attenuation, as well as the underlying causes of radiation-induced color changes [8,9,10]. However, the impact of irradiation on the microstructure of COC remains unclear and requires further research and exploration.

The ethylene-norbornene (E-N) copolymer, which was developed in the late 1950s, falls within this COC category. In 1989, Kaminsky pioneered the synthesis of cyclic olefin polymers notable for their exceptional transparency and high hardness using metallocene catalysts [11]. Different catalysts can facilitate the formation of isotactic or syndiotactic chain structures [12]. By selecting specific catalyst systems, the incorporation of norbornene units can be controlled, allowing for regulation of the copolymer’s microstructure and stereoselectivity [13,14,15,16,17,18,19,20,21,22]. Recent advancements in polymer characterization have incorporated the use of pentads to represent the stereochemistry of consecutive monomer units, offering deeper insights into the spatial arrangements and chirality within COC chains.

**Figure 1:**
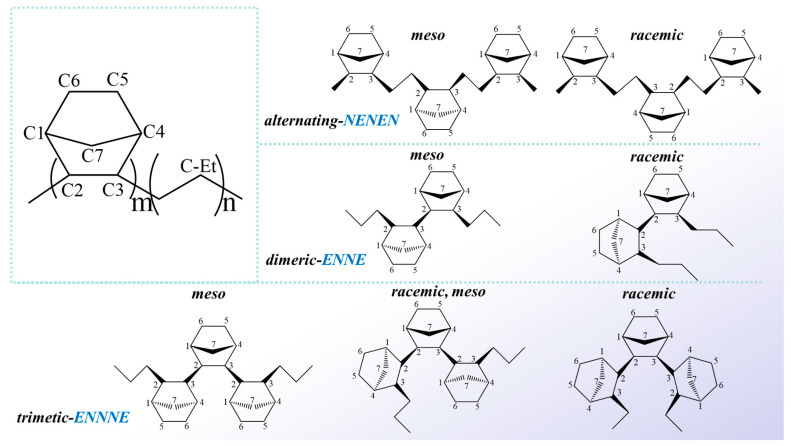
Various segments and microstructures in COC.

In ethylene (E)-norbornene (N) copolymers, the arrangement of norbornene units results in various structural motifs that significantly influence material properties, including alternating (NENEN), dimeric (ENNE), and trimetic (ENNNE) configurations. Recent research, as illustrated in Figure 1, has also explored the presence of meso and racemic structures within COC chains. Meso compounds are notable for their internal symmetry, which renders the optically inactive despite containing multiple chiral centers. These structures arise from symmetrical planes or centers of symmetry within the molecular framework. In contrast, racemic mixtures consist of equal proportions of two enantiomers—mirror-image molecules that do not optically rotate plane-polarized light. The mechanisms by which irradiation induces structural changes in COCs are not yet fully understood. While existing literature provides valuable insights into the structures of COC chain segments, this study aims to investigate the effects of irradiation on COCs based on these findings.

Nuclear Magnetic Resonance (NMR) spectroscopy, encompassing ^1^H, ^13^C, and 2D correlation techniques, is undoubtedly one of the most powerful analytical tools for studying polymer microstructures [23]. NMR enables the characterization of electronic shielding, molecular structure, and the presence of functional groups through the analysis of unique chemical shifts, coupling constants, and relaxation times. The carbon numbering of the utilized norbornene aligns with previous studies [24,25,26,27,28,29,30,31,32,33,34,35,36,37], highlighting differences in distribution and ensuring that C1, C2, and C6 are consistently positioned closer to other norbornene units compared to C3, C4, and C5 [27,28,29,30,31]. The spectral assignments of COCs are notably complex, as each norbornene fragment comprises two stereoisomeric five-membered rings, deviating from the straightforward addition rule of chemical shifts.

^1^H NMR studies [32,33] on COCs with various compositions distinguished hydrogen signal on the ethylene chain segment and on the ring and allowed determination of norbornene content. The norbornene fraction may be calculated using formula (1), where a is the integral sum of the bridging hydrogens (C1-H and C4-H) and b is the rest of the C-H signals, respectively. In addition, the team analyzed the hydrogen atom signals of different environments of COCs using HMQC. The chemical shift analysis of hydrogen is shown in Table 1:

(1) $$\mathrm{Norbornene}\left(\mathrm{mol}\%\right)=2a/(b-2a)$$

Extensive investigations have been conducted on the NMR spectral analysis of ethylene-norbornene copolymers, revealing a significant correlation between the conformational characteristics of these copolymers and their corresponding ^13^C NMR chemical shifts. Notably, critical microstructural features essential for distinguishing isotactic and atactic norbornene sequences were identified and characterized. Furthermore, the influence of monomer concentration on the microstructural evolution of ethylene-norbornene copolymers was systematically investigated. A comprehensive compilation of the ^13^C NMR assignments corresponding to the microstructural elements of COC is also presented in Table 1, providing a detailed reference for structural characterization.

Small-Angle X-ray Scattering (SAXS) is a powerful technique for characterizing the dimensions and morphology of polymer particles and voids, analyzing phase structures in polymer blends, and determining structural features such as long periods, branched chains, molecular chain lengths, and glass transition temperatures. SAXS measures variations in electron density within a material at a scale of 1 to 100 nanometers. When X-rays pass through an ultra-fine powder layer, they are scattered by the electrons in the powder particles, producing scattering at very small angles relative to the incident beam. The intensity distribution of the scattered X-rays correlates with the size and distribution of the particles. The fundamental principle of SAXS relies on the electron density contrast between the scatterers and the surrounding medium. Despite its advancements in elucidating polymer and composite structures, there is a notable lack of research investigating the effects of irradiation on polymer materials, particularly polymer-based composites, using SAXS. For instance, Hai et al. [38] investigated the structural evolution of HDPE/carbon particle-doped HDPE under 1.157 GeV Fe ion irradiation at varying doses using SAXS. The study revealed that carbon particle doping protected the long-period structure of HDPE, conferring certain radiation resistance to the material. Fayolle et al. [39] systematically studied the structural and property evolution of polyoxymethylene under γ-ray irradiation using SAXS and wide-angle X-ray scattering (WAXS). They discovered two competing mechanisms during irradiation: material degradation caused by molecular chain scission and structural reorganization driven by chemical crystallization. At low absorption doses, molecular rearrangement in amorphous regions promoted secondary crystallization, increasing lamellar thickness and significantly enhancing crystallinity. With higher absorption doses, main chain scission became dominant, leading to reduced molar mass, intensified end-group oxidation, and consequent deterioration of mechanical properties. Somani et al. [40] employed synchrotron radiation SAXS to systematically study the shear-induced crystallization process of isotactic PP in supercooled melt. Analysis of SAXS scattering patterns revealed the formation of highly oriented lamellar structures in isotactic PP under shear flow. Rui [41] treated LDPE/carbon nanotube composites with high-energy (170 keV) electron beam irradiation. Porod theory analysis of SAXS data demonstrated that incorporating carbon nanotubes not only enhanced irradiation stability but also mitigated material degradation by suppressing radical migration, in particular showing excellent mechanical property retention at higher absorption doses.

While extensive research has elucidated the irradiation-dependent macroscopic property evolution of COCs, the fundamental microstructural determinants governing these radiation effects remain underexplored at the molecular scale. By employing an integrated approach combining NMR and SAXS, this work systematically investigates the radiation-induced conformational and structural changes in COC chain segments, providing novel mechanistic insights into the deeper understanding of the radiation properties of COC.

## 2. Materials and Methods

### 2.1. Sample Preparation

COCs, specifically TOPAS grades 8007, 5013, 6015, and 6017, were evaluated. The norbornene content in each grade of COC is presented in Table 2. COCs were irradiated at a dose rate of 2 kGy/h at room temperature in air, using a ^60^Co γ-ray source at the Shanghai Institute of Applied Physics. The total absorbed doses for the specimens were 25 kGy and 100 kGy, respectively.

### 2.2. Experimental Operation

Weigh 10–20 mg of the COC sample and dissolve it in deuterated chloroform (CDCl_3_, 99.9% deuterium purity) to prepare a concentrated solution. All ^1^H NMR experiments were conducted at room temperature or 50 °C using a Bruker 500 MHz NMR spectrometer (Berlin, Germany) equipped with a 5 mm probe, with tetramethylsilane (TMS) as the internal standard for calibration. All HSQC and ^13^C NMR experiments were performed at room temperature on a Quantum-I Plus 400 MHz NMR spectrometer (Wuhan, China) with a 5 mm probe, using TMS as the internal standard for calibration. The NMR data were processed and analyzed using MestReNova (15.0) software.

SAXS testing of the sample was performed at the BL10U1 station of the Shanghai Synchrotron Radiation Facility. The two-dimensional SAXS scattering pattern was recorded using an Eiger 1M detector (Dectris, Baden-Daettwil, Switzerland) with a pixel size of 79 μm × 79 μm. The X-ray wavelength was 1.24 A and the distance from the sample to the detector was 4500 mm. The data collection time for all samples was set to 1 s. The SAXS curve, depicting the relationship between scattering intensity I(q) and the scattering vector q = (4π/λ) sinθ, was calculated using Fit 2D (17.006) software, where λ represents the X-ray wavelength and θ denotes the half-scattering angle.

## 3. Results and Discussion

### 3.1. The Effect of Gamma Radiation on the Segments of COC Analyzed via NMR Spectroscopy

**Figure 2:**
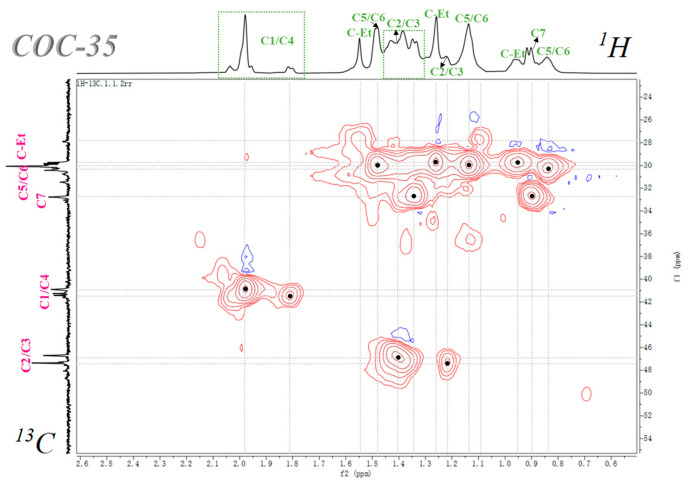
The correspondence between the ^1^H NMR, ^13^C NMR, and HSQC in COC-35. (The spectra were all acquired at room temperature).

^1^H-^13^C HSQC NMR spectroscopy was employed to elucidate structural correlations between protons and directly bonded carbon nuclei in gamma-irradiated COCs. To investigate radiation-induced modifications, the ^1^H and ^13^C NMR spectrum of COC-35 were acquired at ambient temperature and analyzed in conjunction with the 2D ^1^H-^13^C HSQC spectrum, as illustrated in Figure 2, enabling the unambiguous assignment of proton-carbon coupling networks. The key cross-peaks observed in the HSQC spectrum were systematically assigned, as summarized in Table 3, providing critical insights into radiation-induced structural rearrangements at the molecular level.

Subsequent high-temperature NMR measurements significantly improved peak resolution by enhancing molecular chain mobility. Elevated temperatures reduce intermolecular interactions, thereby minimizing peak overlap and broadening effects, which is particularly advantageous for polymer systems. The benefits of high-temperature testing are especially pronounced in polymeric materials, which often exhibit poor solubility or chain entanglement at ambient conditions due to their high molecular weight and complex chain architecture. These factors typically result in weak, broad, or overlapping NMR signals. Heating facilitates solvent penetration and disentanglement of polymer chains, improving sample solubility and promoting more homogeneous molecular dispersion in solution. This leads to enhanced signal intensity and narrower linewidths. Furthermore, increased thermal energy accelerates molecular motion, shortening relaxation times and yielding sharper, more well-defined peaks in the NMR spectra.

**Figure 3:**
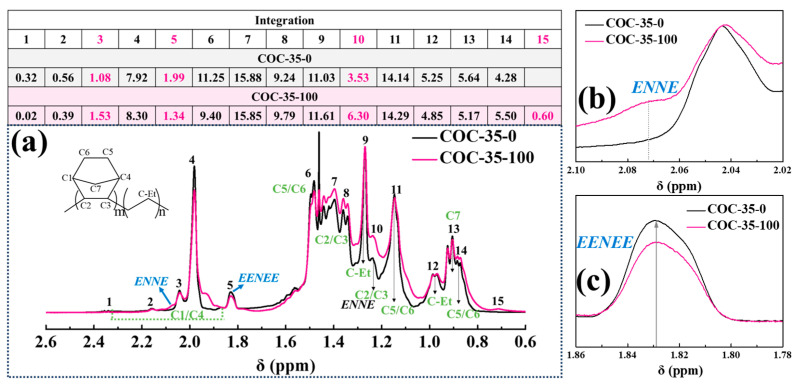
(**a**) Comparative analysis of ^1^H NMR (measured at 50 °C) spectral assignments for COC-35 pre- and post-irradiation. The relative integral area for each signal (normalized to total spectral integral of 100%) is tabulated above the corresponding spectra, with signal variations at 2.07 ppm (**b**) and 1.83 ppm (**c**).

The types of free radicals generated in COCs under γ-ray irradiation were analyzed by electron spin resonance (ESR) spectroscopy. Although varying irradiation doses altered the signal intensities in these materials, they did not change the ESR line shapes, indicating that the radical types were independent of the irradiation dose (as shown in Figure S1). Subsequently, changes in the segmental structures after irradiation were examined using ^1^H NMR spectroscopy: COC-35 is shown in Figure 3, COC-46 in Figure S2, COC-52 in Figure S3, and COC-57 in Figure S4.

For COC-35-100, spectral changes at 2.07 ppm (indicating increased ENNE segment content) and decreased integration area at 1.83 ppm (corresponding to reduced EENEE segments) demonstrate radiation-induced crosslinking of flexible chains, which promotes closer spatial arrangement of the cyclic structures.

**Figure 4:**
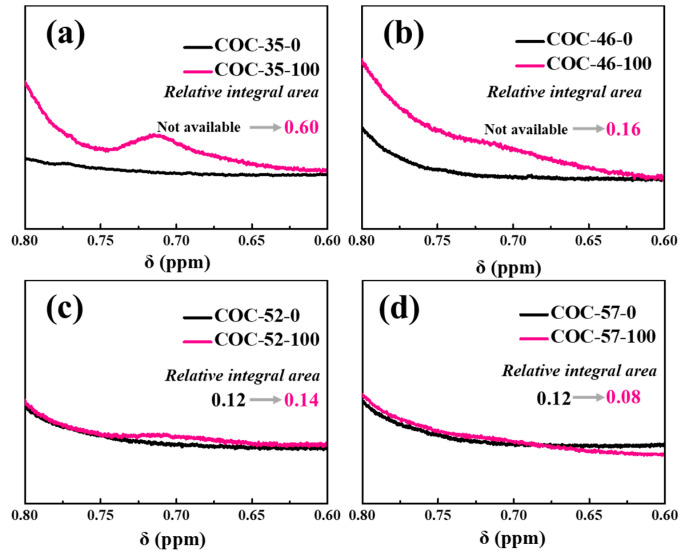
^1^H NMR spectra of (**a**) COC-35, (**b**) COC-46, (**c**) COC-52, and (**d**) COC-57 before and after irradiation: signal variation at 0.71 ppm.

In ethylene-norbornene copolymers, the terminal methyl group (-CH_3_) of the ethylene unit typically appears in the ^1^H NMR spectrum within the range of 0.8–1.2 ppm. However, the presence of a ring structure can cause the chemical shift of the terminal group to move upfield (toward a higher field). For instance, the terminal group near a cyclopropane ring exhibits such an upfield shift [42]. Notably, the appearance of a signal at 0.71 ppm in COC-35-100 and COC-46-100 (refer to Figure 4a,b) suggests that irradiation may induce entanglement of the COC copolymer molecular chains, bringing norbornene units into closer proximity. This proximity could lead to a slight upfield shift of the bridgehead hydrogen signal to 0.71 ppm. Additionally, branching reactions in the flexible segments of the copolymer may also contribute to the appearance of a terminal methyl hydrogen (-CH_3_) signal at 0.71 ppm.

For high-norbornene COC variants, irradiation produces stabilized radicals within sterically hindered chain segments. The restricted mobility of these radicals due to the material’s inherent rigidity prevents efficient recombination, ultimately yielding low-molecular-weight degradation products. This behavior stems from the competing processes of rigid-segment stabilization versus flexible-segment crosslinking, with the balance directly influencing the 0.71 ppm signal intensity. While COC-52-100 exhibits a minor integration increase (0.12 to 0.14), COC-57-100 paradoxically shows reduced intensity (0.12 to 0.08; Figure 4c,d), demonstrating how norbornene content dictates radiation response through steric and mobility effects. Temperature-induced chain mobility also reveals structural modifications through differential scanning calorimetry (DSC) analysis (Figure S5). For irradiated COC-35, the observed increase in *T*_g_ indicates the formation of higher-molecular-weight products that restrict chain mobility. In contrast, systems with higher norbornene content exhibit radiation-induced oxidative degradation, generating lower-molecular-weight fragments. These shorter chains demonstrate greater mobility, manifested by a downward shift in *T*_g_. This dichotomy highlights how composition-dependent radiation responses govern the thermal-mechanical properties of COC materials.

**Figure 5:**
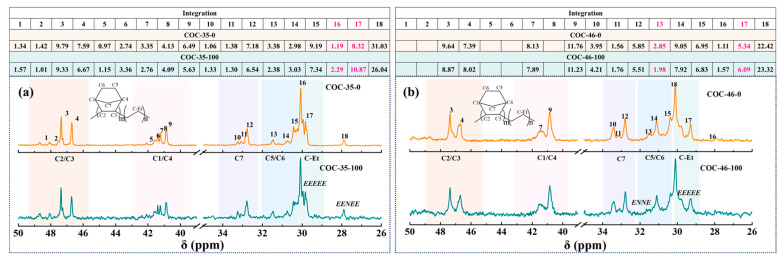
Comparative analysis of ^13^C NMR spectral assignments for COC-35 (**a**) and COC-46 (**b**) pre- and post-irradiation. The relative integral area for each signal (normalized to total spectral integral of 100%) is tabulated above the corresponding spectra.

As the norbornene content increases, the spectral complexity intensifies, potentially leading to overlap in the regions. Longer norbornene sequences may result in signal splitting and shifting, making it challenging to accurately determine the specific content of cyclic structures. To examine the effect of irradiation on the chain structure of COC, this study compared the ^13^C NMR spectra of COCs before and after irradiation at an absorbed dose of 100 kGy, as illustrated in Figure 5. In COC-35-100 (as shown in Figure 5a), the ethylene segment content increased following irradiation. The integral area of C-Et on the EEEEE also increased, indicating that the molecular chains had become twisted and entangled post irradiation, causing the ring structures to move closer together, resulting in stronger peak strengths. 

In the case of sample COC-46 (as shown in Figure 5b), although the signal at ethylene segment showed a slight increased post-irradiation, the ENNE segments decreased.

**Figure 6:**
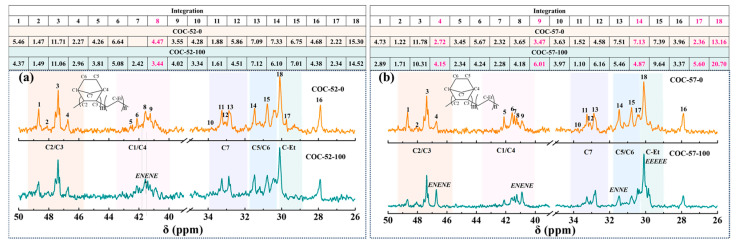
Comparative analysis of ^13^C NMR spectral assignments for COC-52 (**a**) and COC-57 (**b**) pre- and post-irradiation. The relative integral area for each signal (normalized to total spectral integral of 100%) is tabulated above the corresponding spectra.

In Figure 6a, the integral area of NENEN segment in COC-52-100 represented by peak 8 showed an increasing trend. When the norbornene content in COC was increased to 57 mol%, notable changes in peak intensities were observed after irradiation (as shown in Figure 6b). Among these changes, the peak intensities corresponding to the peak 18 and 17 of the ethylene segment showed a significant increase. Similarly, the relative integration areas of the peak 4 in the C2/C3 region and the peak 9 position in the C1/C4 region also increased, suggesting a notable enhancement in the signal intensity of NENEN fragments following irradiation. In contrast, the integration value of the peak 14 position in the C5/C6 region was significantly reduced, indicating a weakening of the signal intensity associated with the ENNE segment. Collectively, these observations imply that the tertiary alkyl radicals within the norbornene ring exhibit limited migratory capacity, resulting in their oxidation or rearrangement into low-molecular-weight products characterized by short-chain segments. This behavior stands in contrast to the irradiation-induced entanglement and branching observed in the flexible chain segments of COC-35-100 and COC-46-100.

These observations collectively demonstrate that the tert-alkyl radicals within the norbornene rings exhibit restricted migration capability, ultimately leading to their oxidation or rearrangement into low-molecular-weight products characterized by short-chain segments. This phenomenon stands in stark contrast to the radiation-induced entanglement and branching observed in the flexible chain segments of COC-35. Furthermore, the findings corroborate earlier FTIR studies showing that increased norbornene content across the four samples significantly enhanced the generation of cyclized radicals upon irradiation [9]. Due to pronounced steric hindrance effects, these radicals displayed constrained mobility and were consequently converted into stable oxidation products, as evidenced by (1) markedly intensified characteristic peaks of carbonyl groups (-C=O) at 1725 cm^−1^ and 1452 cm^−1^ and (2) enhanced post-irradiation signals at 1257 cm^−1^ (tertiary alcohol) and 1095 cm^−1^ (secondary alcohol).

The analysis results of ^1^H NMR and ^13^C NMR indicate that the C-H or C-C bonds in the COC structure break during irradiation, generating alkyl radicals. Due to the significant steric hindrance around the tertiary carbon-centered radicals, the kinetics of their further reactions are limited. In contrast, secondary carbon radicals exhibit higher reactivity because of their lower steric hindrance, making them more prone to coupling reactions with other radicals, which leads to a notable decrease in their signal intensity.

**Figure 7:**
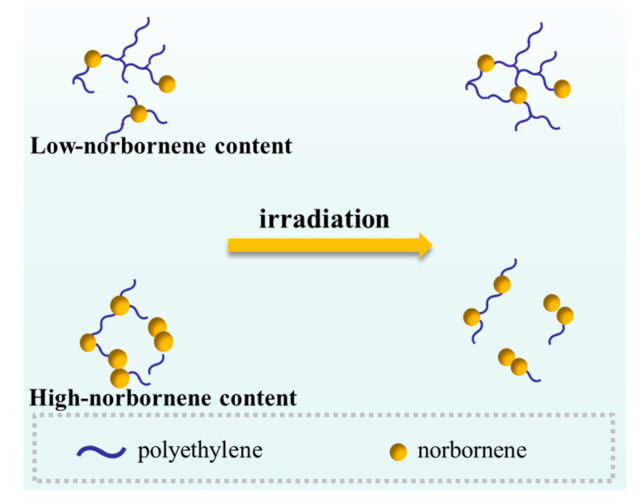
Description of chain segment changes after COC irradiation with different norbornene content.

By comparing the COC structure before and after irradiation using ^1^H NMR and ^13^C NMR, it was found that the flexible chain segments can migrate and form entangled structures after irradiation. As the norbornene content increases, the mobility of the cyclic structures decreases, resulting in a relative reduction in the norbornene dimer structures in COC after irradiation, making the segments more susceptible to degradation (as shown in Figure 7).

### 3.2. The Effect of Gamma Radiation on Particles Inside COC Analyzed by SAXS

**Figure 8:**
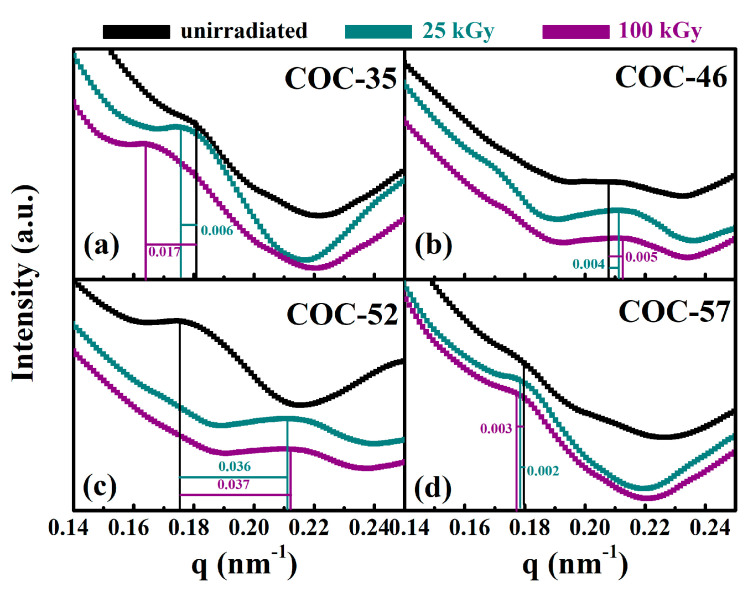
SAXS scattering intensity curves of COC with different norbornene contents. (**a**) COC-35: with norbornene content of 35%; (**b**) COC-46: with norbornene content of 46%; (**c**) COC-52: with norbornene content of 52%; (**d**) COC-57: with norbornene content of 57%.

Additionally, SAXS analysis was employed to compare particle mobility in COC after irradiation. The SAXS diagram of COC exposed to various radiation doses is shown in Figure 8, with the abscissa representing the scattering vector q and the ordinate indicating the scattering intensity I.

The q-value of COC-52 closely resembles those of COC-35 and COC-57, while its irradiated q-value becomes comparable to COC-46. COC-52 contains approximately 52% norbornene, and its molecular structure exhibits a nearly 1:1 ratio between rigid norbornene rings and flexible chain segments. This composition gives rise to a unique semi-loose packing arrangement in the pristine state, where the presence of rigid cyclic units significantly hinders the tight stacking of molecular chains. As a result, the material develops enlarged free volume and pronounced nanoscale heterogeneity—structural characteristics that manifest as a relatively low initial q-value in SAXS measurements.

The relationship between the scattering vector q, the diffraction angle θ, and the wavelength of the incident X-ray is given by q = (4π/λ) sinθ. As the irradiation dose increases, the scattering peak gradually shifts towards larger value of q. Using the Bragg formula: L = 2π/q_max_, the long-period results can be calculated, as shown in Table 1. With increasing dose, the long-period of COC samples slightly decrease. This decrease is attributed to the partial breakage of molecular chains caused by irradiation, particularly in the amorphous regions, resulting in shorter molecular chains that are more prone to rearrangement. Consequently, the statistical average distance, or long period, decreases. In contrast, COC-35, which has a high ethylene content, generates numerous flexible chain segments with free radicals during irradiation. These free radical reactions primarily lead to crosslinking and entanglement of the chain segments, accounting for the observed increasing trend in the long period of COC-35 with the absorbed dose.

The Guinier approximation indicates that at low values of q, the scattering curve can be represented by a specific formula, where *R*_*g*_ is the radius of gyration and *I*_(0)_ represents the intensity at the zero scattering angle (q = 0). To further investigate the structural changes in COC after irradiation, the SAXS curves for COC samples subjected to different absorbed doses were analyzed using the Guinier formula, allowing for the calculation of the radius of gyration (*R*_*g*_) for COC irradiated at various doses. The radius of gyration is defined as the root-mean-square distance between each electron in the particle and its center of mass, providing an indication of the average particle size within the sample. The expression for Guinier’s formula is given in Equation (2):

(2) $$I\left(q\right)=I_{e}Mn^{2}\exp\left(-\frac{\lambda^{2}R_{g}^{2}}{3}\right)$$

The exponential term in the Guinier approximation, derived from linearly fitting ln(I) against q^2^*R*_*g*_^2^, is known as the Guinier plot. The specific region selected for this linear fitting is called the “Guinier region”. The *R*_*g*_ value is calculated from the slope of the fitted line (*k*) using Equation (3):

(3) $$R_{g}=\sqrt{\left(-3k\right)}$$

**Figure 9:**
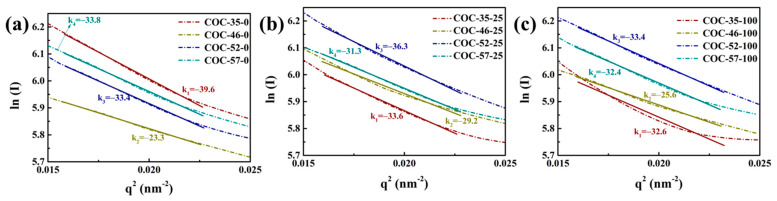
Guinier curves of COC with different norbornene content (**a**–**c**).

Figure 9 presents the Guinier plot of COC samples that have been irradiated at various doses. As the absorbed dose increases, the absolute value of the slope of the fitted line decreases. The calculated results of gyration are summarized in Table 4. Among the four COCs, sample COC-35 exhibits the highest *R*_*g*_ value. This implies that the entanglement of cross-linked structures in COC-35 causes a decrease in the average size of the internal particles and an increase in the mean statistical distance.

**Figure 10:**
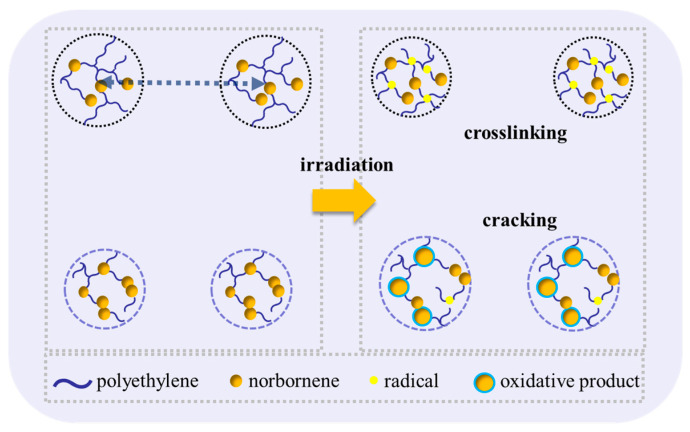
Relationship between norbornene content and microstructure in COC during irradiation.

As the absorbed dose increases, the content of tertiary alkyl radicals generated by cleavage of chemical bonds in COC-46, COC-52, and COC-57 also increases after irradiation. These radicals have restricted mobility and are less prone to recombination. Instead, they can react with oxygen to form minor oxidation products. As a result, this leads to an increase in the average size of the internal particles and a decrease in the mean statistical distance. Figure 10 summarizes the structural changes in the COC chain segments, including the long-period and radius of gyration models of the internal particles after irradiation.

In COC-35-100 and COC-46-100 systems with low norbornene content, the emergence of a characteristic signal at 0.71 ppm in NMR spectra reveals two distinct radiation-induced mechanisms. High-energy irradiation may trigger molecular chain crosslinking, bringing norbornene units into closer proximity and altering the electronic environment of bridge hydrogens, thereby shifting their chemical displacement upfield to 0.71 ppm. Simultaneously, γ-ray-induced radical reactions could promote branching and crosslinking in flexible chain segments, with newly formed terminal methyl protons overlapping with the shifted bridge hydrogens at 0.71 ppm. These chemical transformations correlate closely with the structural evolution observed by SAXS. For instance, COC-35 initially exhibits the largest *R*_g_, but irradiation-induced crosslinking reduces particle size, which directly corresponds to the enhanced peak at 0.71 ppm in NMR spectra. In contrast, COC-46 undergoes oxidation due to the limited recombination of tertiary alkyl radicals generated by chain scission, leading to an increased *R*_g_, while no significant enhancement in crosslinking signal is detected by NMR. For high-norbornene-content systems (COC-52-100 and COC-57-100), steric hindrance in rigid segments severely restricts radical migration and recombination, making β-scission the dominant degradation pathway. This manifests weakly in NMR spectra as minimal or even slightly reduced signal intensity at 0.7 ppm for COC-57, while SAXS shows a general increase in *R*_g_, reflecting expansion of particulate dimensions due to oxidative degradation of the backbone. The combined NMR and SAXS analysis demonstrates that chain scission in rigid segments directly contributes to mesoscale structural expansion. In amorphous resins, changes in spatial-scale density fluctuations detected by SAXS correlate with chemical bond modifications revealed by NMR. For COCs, irradiation-induced chain scission shortens amorphous chains and may induce rearrangement, reducing long-period spacing. In ethylene-rich COC-35, however, crosslinking between flexible segments dominates, increasing long-period spacing instead. These structural changes find molecular-level correspondence: crosslinking networks are evidenced by altered bridge hydrogen environments in NMR, while chain scission is reflected in decreased peak intensity. By integrating NMR and SAXS observations, a clear pattern emerges: Low-norbornene COCs primarily undergo radical migration-mediated crosslinking and particle contraction, whereas high-norbornene COCs degrade predominantly via β-scission-induced backbone dissociation and particle expansion. This structure–degradation relationship elucidates the critical role of chemical composition in determining radiation stability.

## 4. Conclusions

Gamma irradiation induces free radical formation and migration in COCs, leading to significant polymer chain reorganization. Combined ^1^H and ^13^C NMR analysis reveals enhanced entanglement of flexible chain segments in COC-35. Conversely, COC samples containing 57 mol% norbornene exhibit attenuated multicomponent signals in NMR spectra, indicating that radical mobility is substantially restricted within the rigid cyclic structures, ultimately leading to chain scission. SAXS data corroborate these findings, demonstrating post-irradiation chain entanglement and—notably for high-norbornene COCs—increased particle size caused by radiation-induced oxidation products.

Collectively, these NMR and SAXS analyses systematically elucidate how the norbornene content dictates the radiation-induced structural evolution in COCs, thus refining our understanding of the effects of gamma radiation on COCs with different norbornene compositions.

## Figures and Tables

**Table 1:** ^1^H NMR and ^13^C NMR assignments of COC microstructures, including alternating-NENEN, dimeric-ENNE, and trimetic-ENNNE configurations.

Sequences		C Chemical Shifts	Reference	H Chemical Shifts	Reference
C-Et	EEEEE	27.7	[27,28,29,30,31,32,33,34,35,36,37]	0.9–1.5	
	NENEN	28.0			
C5/C6	ENNE	26.2	[27,31,32,33,34,35,36,37]	0.9–1.5	
	ENNE	29.7	[27,31,32,33,34,35,36]		
	NENEN	28.3	[27,31,32,33,34,35,36]		
C7	NENEN	31.0	[27,31,32,33,34,35,36]	0.9	
	ENNE	31.3	[27,31,32,33,34,35,36]	1.4	
C1/C4	EENEE	39.5	[27,31,32,33,34,35,36]	Not available	[32,33]
	NENEN	39.5	[27,31,32,33,34,35,36]	1.9	
	ENNE	40.4	[27,31,32,33,34,35,36]	1.8, 2.0	
C2/C3	NENEN	45.2	[27,28,29,30,31,32,33,34,35,36]	1.4	[32,33]
	ENNE	48.1	[27,28,29,30,31,32,33,34,35,36]	1.2, 1.4	
	NENNN	50.8–52.0	[27,34,36]	Not available	

**Table 2:** Content of norbornene in each COC grade.

TOPAS Grades	Norbornene/mol% (Reference)	Norbornene/mol% (Sample)	Absorbed Dose (kGy)	Sample
8007	35	37.5	25	COC-35-25
			100	COC-35-100
5013	46	48.3	25	COC-46-25
			100	COC-46-100
6015	52	52.5	25	COC-52-25
			100	COC-52-100
6017	57	55.5	25	COC-57-25
			100	COC-57-100

**Table 3:** Attribution of cross peaks in ^1^H-^13^C HSQC spectrum of COC-35.

	H Chemical Shifts	C Chemical Shifts	Sequences	Reference
C-Et	1.0	29.6	EENEE	
	1.1	29.9	NENEN	This work
	1.6	27.7	ENNE	
C5/C6	0.8	30.2	EENEE	This work
	1.1	29.9	NENEN	
	1.5	29.9	ENNE	
C7	0.9	32.5	EENEE	This work
	1.3	32.5	ENNE	
C1/C4	1.8	41.5	NENEN/EENEE	[24]
	2.0	40.9	ENNE	
C2/C3	1.2	47.3	ENNE	[24]
	1.4	47.0	NENEN/EENEE	
	1.4	48.6	ENNE	

**Table 4:** Internal structural parameters of COC before and after irradiation.

Sample		Long Period (nm)	*R*_*g*_ (nm)
Norbornene 35 mol%	COC-35-0	35.2	10.9
	COC-35-25	35.4	10.0
	COC-35-100	38.1	9.9
Norbornene 46 mol%	COC-46-0	29.8	8.4
	COC-46-25	29.5	9.4
	COC-46-100	29.5	8.8
Norbornene 52 mol%	COC-52-0	35.1	10.0
	COC-52-25	29.6	10.4
	COC-52-100	29.5	10.0
Norbornene 57 mol%	COC-57-0	35.4	8.4
	COC-57-25	34.9	9.7
	COC-57-100	34.9	9.9

## Data Availability

The original contributions presented in the study are included in the article/Supplementary Materials; further inquiries can be directed to the corresponding authors.

## Supplementary Materials

The following supporting information can be downloaded at: https://www.mdpi.com/article/10.3390/polym17131751/s1, Figure S1. ESR spectra of COCs with different norbornene contents, HDPE, and COP after irradiation: (a) absorbed dose of 25 kGy, (b) absorbed dose of 100 kGy. (c) Types of free radicals generated by irradiation in COCs with varying norbornene contents, HDPE, and COP; Figure S2. Comparative analysis of 1H NMR (measured at 50 ℃) spectral assignments for COC-46 pre- and post-irradiation. The relative integral area for each signal (normalized to total spectral integral of 100%) is tabulated above the corresponding spectra; Figure S3. Comparative analysis of 1H NMR (measured at 50 ℃) spectral assignments for COC-52 pre- and post-irradiation. The relative integral area for each signal (normalized to total spectral integral of 100%) is tabulated above the corresponding spectra; Figure S4. Comparative analysis of 1H NMR (measured at 50 ℃) spectral assignments for COC-52 pre- and post-irradiation. The relative integral area for each signal (normalized to total spectral integral of 100%) is tabulated above the corresponding spectra; Figure S5. DSC curves of (a) COC-35, (b) COC-46, (c) COC-52, and (d) COC-57: comparison between pre- and post-irradiation states. References [43,44,45] are cited in Supplementary Materials.
