# Supplementary material for: Gamma Irradiation-Induced Changes in Microstructure of Cyclic Olefin Copolymer (COC) Revealed by NMR and SAXS Characterization
Source: Polymers (Basel). 2025 Jun 24;17(13):1751. doi: 10.3390/polym17131751 (PMC12251773; doi:10.3390/polym17131751)
Supplement: Supplementary file 1 [file polymers-17-01751-s001.zip › polymers-3661698-supplementary.pdf]

## No: polymers-3661698

## SUPPORTING INFORMATION

# Gamma Irradiation-Induced Changes in Microstructure of Cyclic Olefin Copolymer (COC) Revealed by NMR and SAXS Characterization

**Fan Zhang [1,2], Heng Lei [1,2,3], Feng Guo [1,2], Jiangtao Hu [1,2], Haiming Liu [3], Qing Wang [3], Weihua Liu [1,2], Zhe Xing [1,2,*], Guozhong Wu [1,2,3,*]**

1 Shanghai Institute of Applied Physics, Chinese Academy of Sciences, No. 2019 Jialuo Road, Jiading District, Shanghai 201800, China

2 University of Chinese Academy of Sciences, Beijing 100049, China

3 School of Physical Science and Technology, ShanghaiTech University, Shanghai 201210, China

* Corresponding authors: Zhe Xing (Tel.: +8621-39194630; email: xingzhe@sinap.ac.cn), Guozhong Wu (Tel.: +8621-39194531; email: wuguozhong@sinap.ac.cn)

**Experimental operation:**

**Electron Spin Resonance (ESR):** The initial ESR spectra were recorded under the following instrument parameters: microwave frequency 9600 MHz, microwave power 0.001 W, NMR frequency 100 kHz, central magnetic field 338 mT, sweep width ±25 mT, time constant 0.03 ms, scan time 1 min, modulation frequency 100 kHz, and modulation amplitude 0.35 mT.

**Differential scanning calorimetry (DSC):** The measurements were performed using a Mettler DSC 3 with a temperature range of 30-240℃, a heating rate of 10℃/min, and a nitrogen purge flow rate of 50 mL/min.

**Results and Discussion:**

We propose that radical reactions are responsible for the observed structural changes. By comparing the ESR spectra of irradiated COP and HDPE, we analyzed the radical species generated in COC upon $\gamma$-irradiation. As shown in **Figure S1**, HDPE exhibits a characteristic quintet pattern from alkyl radicals alongside mixed signals from allyl and polyenyl radicals. Notably, both HDPE and COP display relatively simple radical compositions with symmetrical ESR signals [1-3]. While varying absorbed doses altered signal intensities in these materials, they did not change the ESR line shapes, indicating dose-independent radical types.

In contrast, $\gamma$-irradiated COCs with different norbornene contents showed gradual variations in ESR line shapes with increasing cyclic structure content. This evolution suggests compositional changes in the radical population. From COC-35 to COC-57, the growing number of tertiary carbon sites linking flexible segments to cyclic structures leads

to higher relative proportions of tertiary alkyl radicals.

Our comprehensive analysis reveals that γ-irradiation generates abundant alkyl radicals in COC systems. While secondary carbon radicals in flexible segments demonstrate high mobility for radical recombination, materials with higher norbornene contents produce increased amounts of tertiary alkyl radicals. The restricted chain mobility imposed by rigid cyclic structures slows radical migration, making these tertiary species more susceptible to subsequent oxygen reactions during room-temperature storage.

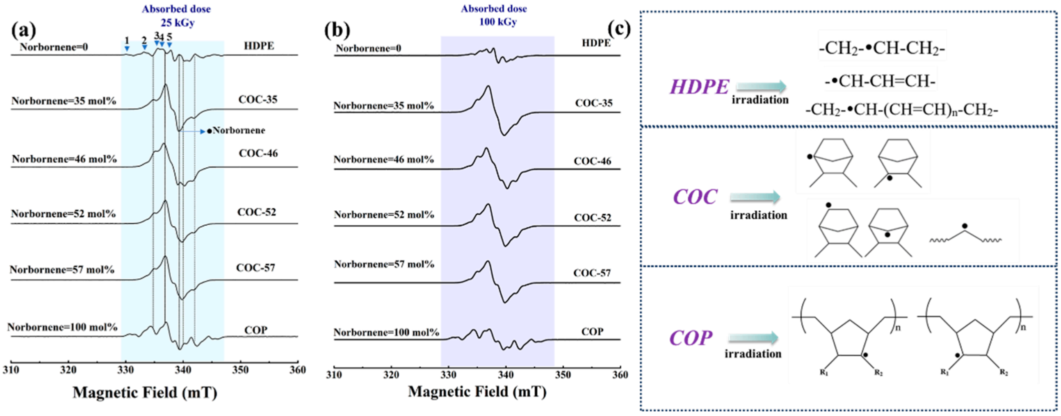


**Figure S1**. ESR spectra of COCs with different norbornene contents, HDPE, and COP after irradiation: (a) absorbed dose of 25 kGy, (b) absorbed dose of 100 kGy. (c) Types of free radicals generated by irradiation in COCs with varying norbornene contents, HDPE, and COP.

| Integration | | | | | | | | | | | | | | |
|---|---|---|---|---|---|---|---|---|---|---|---|---|---|---|
| 1 | 2 | 3 | 4 | 5 | 6 | 7 | 8 | 9 | 10 | 11 | 12 | 13 | 14 | 15 |
| COC-46-0 | | | | | | | | | | | | | | |
| 0.19 | 3.18 | 3.15 | 6.72 | 0.98 | 13.69 | 10.46 | 11.21 | 3.80 | 3.84 | 17.26 | 5.72 | 3.60 | 4.30 | |
| COC-46-100 | | | | | | | | | | | | | | |
| 0.26 | 3.25 | 3.05 | 6.61 | 1.36 | 11.58 | 9.47 | 15.90 | 4.32 | 4.08 | 16.42 | 6.95 | 5.92 | 6.34 | 0.16 |

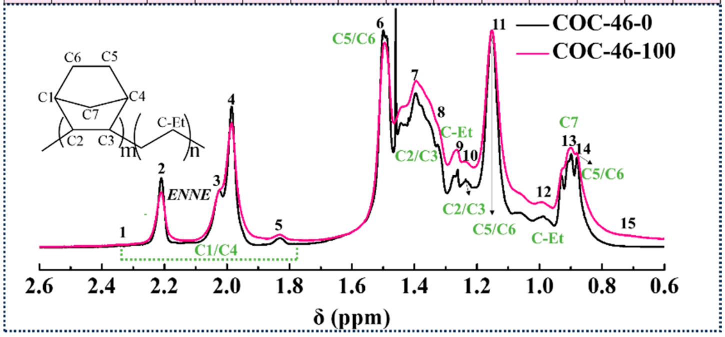


**Figure S2**. Comparative analysis of $^1$H NMR (measured at 50℃) spectral assignments for COC-46 pre- and post-irradiation. The relative integral area for each signal (normalized to total spectral integral of 100%) is tabulated above the corresponding spectra.

The $^1$H NMR spectra of COC-52 and COC-57 (**Figures S3** and **S4**, respectively) exhibit two new signals at 1.97 and 1.77 ppm compared to COC-35 and COC-46.

The signal at 1.97 ppm and Peak 4 both originate from ***ENNE***, with the former assigned to the ***meso***-conformation due to its lower chemical shift. Similarly, the upfield signal at 1.77 ppm and Peak 5 are attributable to ***EENEE***, where the lower chemical shift of the 1.77 ppm resonance also supports a ***meso***-configuration.

| Integration | | | | | | | | | | | | | | |
|---|---|---|---|---|---|---|---|---|---|---|---|---|---|---|
| 1 | 2 | 3 | 4 | 5 | 6 | 7 | 8 | 9 | 10 | 11 | 12 | 13 | 14 | 15 |
| COC-52-0 | | | | | | | | | | | | | | |
| 0.61 | 1.86 | 2.75 | 6.68 | 3.47 | 13.91 | 15.87 | 5.98 | 3.08 | 6.32 | 14.66 | 3.48 | 6.20 | 5.05 | 0.12 |
| COC-52-100 | | | | | | | | | | | | | | |
| 0.71 | 1.88 | 2.71 | 7.06 | 3.52 | 13.61 | 14.74 | 6.20 | 2.58 | 6.40 | 15.14 | 3.01 | 6.43 | 5.63 | 0.14 |

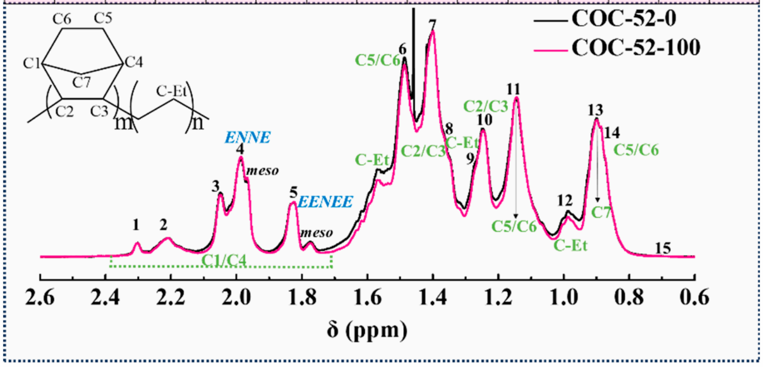


**Figure S3**. Comparative analysis of $^{1}$H NMR (measured at 50 ℃) spectral assignments for COC-52 pre- and post-irradiation. The relative integral area for each signal (normalized to total spectral integral of 100%) is tabulated above the corresponding spectra.

| Integration | | | | | | | | | | | | | | |
|---|---|---|---|---|---|---|---|---|---|---|---|---|---|---|
| 1 | 2 | 3 | 4 | 5 | 6 | 7 | 8 | 9 | 10 | 11 | 12 | 13 | 14 | 15 |
| COC-57-0 | | | | | | | | | | | | | | |
| 0.53 | 1.71 | 2.41 | 6.90 | 4.01 | 10.90 | 14.28 | 7.13 | 3.50 | 6.27 | 14.66 | 3.54 | 5.43 | 5.99 | 0.12 |
| COC-57-100 | | | | | | | | | | | | | | |
| 0.50 | 1.63 | 2.60 | 6.87 | 3.31 | 13.35 | 14.83 | 6.80 | 4.24 | 5.48 | 15.50 | 3.29 | 5.41 | 6.85 | 0.08 |

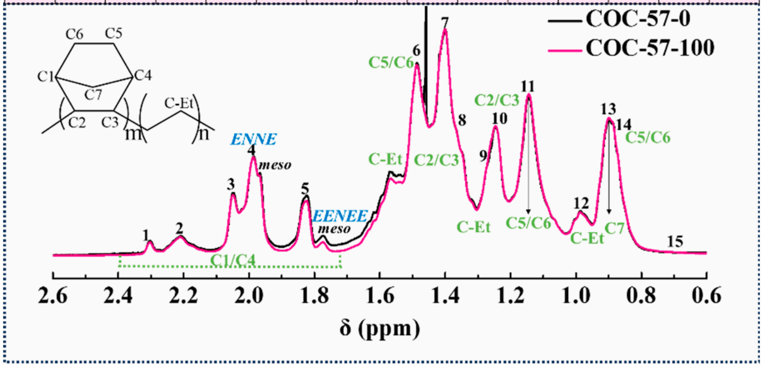


**Figure S4**. Comparative analysis of $^{1}$H NMR (measured at 50 ℃) spectral assignments for COC-52 pre- and post-irradiation. The relative integral area for each signal (normalized to total spectral integral of 100%) is tabulated above the corresponding spectra.

Temperature-induced chain mobility also reveals structural modifications through DSC analysis (**Figure S5**). For irradiated COC-35, the observed increase in $T_g$ indicates the formation of higher molecular weight products that restrict chain mobility. In contrast, systems with higher norbornene content exhibit radiation-induced oxidative degradation, generating lower molecular weight fragments. These shorter chains demonstrate greater mobility, manifested by a downward shift in $T_g$. This dichotomy highlights how composition-dependent radiation responses govern the thermal-mechanical properties of COC materials.

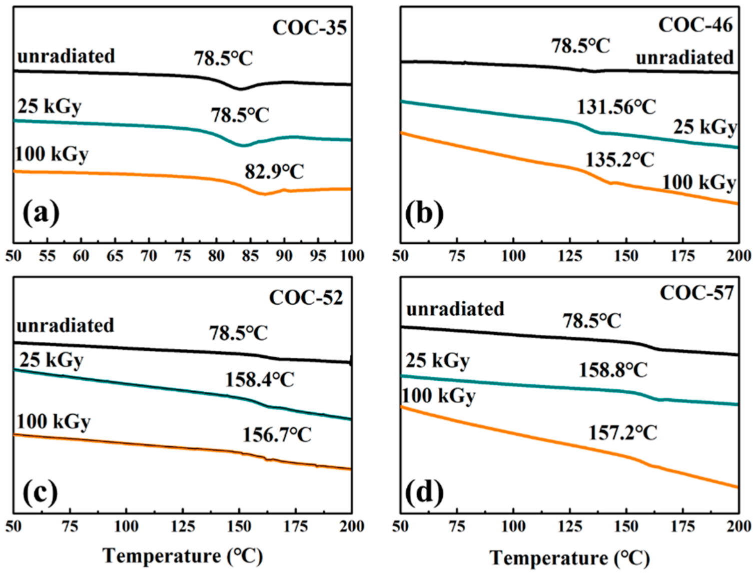


**Figure S5**. DSC curves of (a) COC-35, (b) COC-46, (c) COC-52, and (d) COC-57: comparison between pre- and post-irradiation states.

## References

[1] Zhao Y, Wang M, Tang Z, et al. ESR study of free radicals in UHMW-PE fiber irradiated by gamma rays. Radiation Physics and Chemistry, 2010, 79(4): 429-433.

[2] Kiminami H, Imae Y, Takahashi E, et al. Electron beam sterilization of cyclo olefin polymer leads to polymer degradation and production of alkyl radicals. Journal of Applied Polymer Science, 2016, 133(23), 43498.

[3] Mochizuki A, Ono D, Kiminami H, et al. Carbon radicals generated by solid polymers: Electron spin resonance spectroscopy for detection of species in water. Journal of Applied Polymer Science, 2019, 137(17), 48604.
